# Identification of the Functional Domain of Thyroid Hormone Receptor Responsible for Polychlorinated Biphenyl–Mediated Suppression of Its Action *in Vitro*

**DOI:** 10.1289/ehp.11176

**Published:** 2008-05-14

**Authors:** Wataru Miyazaki, Toshiharu Iwasaki, Akira Takeshita, Chiharu Tohyama, Noriyuki Koibuchi

**Affiliations:** 1 Department of Integrative Physiology, Gunma University Graduate School of Medicine, Maebashi, Japan; 2 Endocrine Center, Toranomon Hospital and Okinaka Memorial Institute for Medical Research, Tokyo, Japan; 3 Laboratory of Environmental Health Sciences, Center for Disease Biology and Integrative Medicine, Graduate School of Medicine, University of Tokyo, Tokyo, Japan

**Keywords:** dioxin, DNA-binding domain, polychlorinated biphenyl, thyroid hormone receptor, transcription

## Abstract

**Background:**

Polychlorinated biphenyls (PCBs), polychlorinated dibenzo-*p*-dioxins, and poly-chlorinated dibenzofurans adversely affect the health of humans and various animals. Such effects might be partially exerted through the thyroid hormone (TH) system. We previously reported that one of the hydroxylated PCB congeners suppresses TH receptor (TR)-mediated transcription by dissociating TR from the TH response element (TRE). However, the binding site of PCB within TR has not yet been identified.

**Objectives:**

We aimed to identify the functional TR domain responsible for the PCB-mediated suppression of TR action by comparing the magnitude of suppression using several representative PCB/dioxin congeners.

**Materials and methods:**

We generated chimeric receptors by combining TR and glucocorticoid receptor (GR) and determined receptor-mediated transcription using transient transfection-based reporter gene assays, and TR-TRE binding using electrophoretic mobility shift assays.

**Results:**

Although several PCB congeners, including the hydroxylated forms, suppressed TR-mediated transcription to various degrees, 2,3,7,8-tetrachlorodibenzo-*p*-dioxin did not alter TR action, but 2,3,4,7,8-pentachlorodibenzofuran weakly suppressed it. The magnitude of suppression correlated with that of TR–TRE dissociation. The suppression by PCB congeners was evident from experiments using chimeric receptors containing a TR DNA-binding domain (DBD) but not a GR-DBD.

**Conclusions:**

Several nondioxin-like PCB congeners and hydroxylated PCB compounds suppress TR action by dissociating TR from TRE through interaction with TR-DBD.

Polychlorinated biphenyls (PCBs), poly-chlorinated dibenzo-*p*-dioxins (PCDDs), and polychlorinated dibenzofurans (PCDFs) are extremely persistent environmental compounds that adversely affect the health of humans and other animals. These compounds are toxic to the fetal and early post-natal developing brain, which is exposed via the placenta and breast milk as a result of maternal exposure (Gladen and Rogan 1991; Safe 1994; Tilson et al. 1990; Tilson and Kodavanti 1997), even if the exposure level is too low to induce maternal toxicity (Chen et al. 1992; Jacobson and Jacobson 1996a; Jacobson et al. 1985, 1990). Jacobson and Jacobson (1996b) suggested that exposure to PCBs *in utero* induced intellectual impairment in children born to mothers who consumed excessive amounts of sport fish obtained from the Great Lakes area in the United States. Disruption of cognitive development among children exposed to dioxins and PCBs has been documented in accidental human exposures, such as in the Yusho and Yu-cheng incidences (Aoki 2001), and confirmed in experimental animals (Giesy and Kannan 1998; Jacobson and Jacobson 1997). In addition, exposure to PCBs may alter dendrito-genesis in several brain regions during development (Kimura-Kuroda et al. 2005; Lein et al. 2007).

The effects of PCBs/dioxins on the brain have been interpreted in several ways. First, dioxin-like PCB congeners are able to bind to and activate aryl hydrocarbon receptors (AhRs), exerting various toxic effects. The degree of such effects is numerically expressed as the toxicity equivalency factor (TEF) and is standardized to 2,3,7,8-tetrachlorodibenzo-*p*-dioxin (TCDD; TEF = 1) (Van den Berg et al. 2006). However, the TEF concept might not fully encompass the developmental neurotoxicity of PCBs, because AhR expression in the brain may be regional (Huang et al. 2000; Petersen et al. 2000) and because PCB congeners are considered to have neuro-toxicities via both AhR-dependent and AhR-independent mechanisms (Giesy and Kannan 1998; Tilson and Kodavanti 1997). Second, PCBs may disrupt intracellular signaling pathways that are essential not only for brain function but also for brain development (reviewed by Kodavanti 2005), which will disturb intra-cellular calcium homeostasis (Kodavanti et al. 1993). For example, *ortho*-substituted non-coplanar congeners might alter protein kinase C translocation, cellular dopamine uptake, and the formation of reactive oxygen species. Such toxicity is referred to as the neurotoxicity equivalent (reviewed by Kodavanti 2005; Simon et al. 2007).

In addition to these signaling pathways, we and several other groups have focused on the possible interactions of these chemicals with the thyroid hormone (TH) system (Bogazzi et al. 2003; Iwasaki et al. 2002; Kitamura et al. 2005; Miyazaki et al. 2004). TH is crucial for brain development, and TH deficiency during the critical perinatal period has been reported to cause cretinism, with severe cognitive and/or mental disorders in the offspring (Koibuchi and Chin 2000; Oppenheimer and Schwartz 1997; Porterfield and Hendrich 1993). PCB/dioxin congeners are considered to cause neurotoxicity by altering TH homeo-stasis in the developing brain. Some researchers have reported that exposure to PCB congeners results in thyroid enlargement and reduced serum total thyroxine (T_4_) levels with normal levels of triiodothyronine (T_3_), an active compound of TH (Brouwer et al. 1998; Hauser et al. 1998; Porterfield 2000), during a possible critical period of TH action. Certain PCB congeners were reported to induce the expression of a microsomal enzyme, uridine diphosphate glucuronosyl-transferase, which glucuronizes T_4_ to facilitate excretion (Hood et al. 2003; Liu et al. 1995). Exposure to TCDD also results in morphologic and functional alterations in the thyroid of adult rodents (Gorski and Rozman 1987; Henry and Gasiewicz 1987; Potter et al. 1986; Van Birgelen et al. 1995). Such exposure induces not only an increase in the volume of thyroid follicular cells, followed by hyperplasia, but also follicular thyroid tumors in rats (Huff et al. 1991; Sewall et al. 1995). Both PCDD and PCDF induce T_3_ and T_4_ excretion, thereby decreasing plasma T_3_ and T_4_ levels (Bastomsky 1977; Kohn et al. 1996; Nishimura et al. 2002). These results indicate that PCBs/dioxins disrupt the TH system by decreasing blood TH levels, which in turn induces hypothyroidism in various organs.

Perinatal exposure to PCBs/dioxins in laboratory animals induces a decrease in plasma T_4_ levels without significantly altering the growth (Lein et al. 2007; Zoeller et al. 2000), and T_3_ levels remain within the normal range (Porterfield 2000), indicating that the toxicity of these chemicals does not manifest by altering blood TH levels. On the other hand, because the molecular structures of PCBs/dioxins are similar to those of TH, these chemicals are considered to act via TH receptors (TRs) (McKinney 1989). Furthermore, these compounds can be transferred across the blood–brain barrier and accumulate in the brain (Brouwer and van den Berg 1986; Cheek et al. 1999; Darnerud et al. 1996; McKinney 1989). These findings suggest that PCBs/dioxins induce abnormal brain development by directly acting on TR.

We therefore performed a series of experiments and found that a hydroxylated (OH) PCB compound [4-OH-2′,3,3′,4′,5′-penta-chlorobiphenyl (pentaCB); 4-OH-PCB-106] at a concentration of 10^−10^ M suppresses TR-mediated transcription induced by TH (Iwasaki et al. 2002). The magnitude of suppression induced by 4-OH-PCB-106 was cell-type dependent and most obvious in clonal TE671 cells derived from human cerebellar granule cells (Iwasaki et al. 2002), and this suppression was due to the partial dissociation of TR from the TH response element (TRE) (Miyazaki et al. 2004). These results suggest that PCBs directly act on TR, although the TR functional domain responsible for PCB action remains obscure.

Here, we report that the magnitude of the suppression of TR-mediated transcription differs among a variety of congeners of PCBs/dioxins. In addition, we identified the functional domain responsible for PCB action using chimeric receptors generated from TR and the glucocorticoid receptor (GR).

## Materials and Methods

### Chemicals

We purchased T_3_ from Sigma Chemical Co. (St. Louis, MO, USA), and TCDD and PCDFs [2,3,4,7,8-pentachloro-dibenzofuran (pentaCDF), 2,8-diCDF, and 2-monoCDF] from Cambridge Isotope Laboratory (Andover, MA, USA); all congeners were > 98% pure. We purchased all PCB congeners [3,3′,4,4′-tetraCB (PCB-77), 3,3′,4,4′,5-pentaCB (PCB-126), 2,3,4,4′,5-pentaCB (PCB-114); 2,3′,4,4′,5-pentaCB (PCB-118); and 2,2′,4,4′,5,5′-hexaCB (PCB-153)] and OH-PCBs [4-OH-2′,3,3′,4′,5′-pentaCB (4-OH-PCB-106); 4-OH-2,3,3′,4,5,5′-hexaCB (4-OH-PCB-159); 4-OH-2,3,3′,5,5′,6-hexaCB (4-OH-PCB-165); and 4-OH-2,2′,3,4′,5,5′,6-heptaCB (4-OH-PCB-187)] from AccuStandard Chemicals (New Haven, CT, USA). All PCB congeners were > 99% pure, and OH-PCBs were > 98% pure.

### Plasmids

The expression vectors for human TRβ1, GR, and mouse retinoid X receptor β (RXRβ), as well as the 2× glucocorticoid response element-luciferase (LUC) reporter in pTAL-LUC (BD Biosciences Clontech, Palo Alto, CA, USA) are described elsewhere (Iwasaki et al. 2001). The LUC reporter constructs, the chick lysozyme (F2)–thymidine kinase (TK)-LUC, and the artificial direct repeat TRE, direct repeat 4 (DR4)-TK-LUC, in the PT109 vector are also described elsewhere (Koibuchi et al. 1999).

We subcloned restriction enzyme fragments of the cDNA inserts of human TRβ1 and GR into the *Kpn*I and *Xba*I sites of pcDNA3. To create human TRβ1 with *Not*I and *Xho*I sites, we changed the oligo-nucleotides used to create the *Not*I site from Asp-97 to Arg, from Lys-98 to Pro, and from Asp-99 to Pro. The oligonucleotides we used to create the *Xho*I site were changed from Thr-171 to Leu and from Asp-172 to Gly (Thompson and Evans 1989). The creation of the *Not*I site on GR changed Pro-416 to Arg, whereas the creation of the *Xho*I site did not alter the GR amino acid sequence.

We constructed chimeric receptors by exchanging *Kpn*I*-Not*I, *Not*I*-Xho*I, or *Xho*I*-Xba*I restriction fragments of human TRβ1 and GR with *Not*I and *Xho*I sites.

### Cell culture

We maintained CV-1 and TE671 cells in Dulbecco’s modified Eagle’s medium supplemented with 5 μg/mL penicillin/streptomycin and 10% fetal bovine serum deprived of small lipophilic hormone at 37°C under a 5% CO_2_ atmosphere as previously described (Iwasaki et al. 2002).

### Transient transfection-based reporter gene assays

We plated cells in 24-well plates 2 days before transfection by calcium phosphate coprecipitation (Iwasaki et al. 2002). The internal control was a cytomegalovirus–β-galactosidase plasmid. Sixteen to 24 hr later, the cells were incubated for 24 hr in fresh medium containing T_3_ and/or PCBs/dioxins. We then harvested the cells to measure the LUC activities as previously described (Iwasaki et al. 2002). We balanced total amounts of DNA per well by adding pcDNA3 plasmids (Invitrogen, San Diego, CA, USA). LUC activities were normalized to that of β-galactosidase and then calculated as relative LUC activity. We repeated all transfection studies at least twice in triplicate. Data represent means ± SEs of triplicates. We analyzed the data by analysis of variance (ANOVA) and by post hoc comparisons using Bonferroni’s multiple range test.

### Electrophoretic mobility shift assay

The methods for the electrophoretic mobility shift assay (EMSA) have been previously described (Satoh et al. 1996). Briefly, we labeled double-stranded oligonucleotides using the Klenow fragment with [α-32P]-dCTP. We incubated *in vitro* transcribed and translated human TRβ1, mouse RXRβ, and 1 × 104 counts per minute labeled nucleotides in binding buffer [25 mM HEPES (pH 7.6), 5 mM MgCl_2_, 4 mM EDTA, 110 mM NaCl, 5 μg/μL bovine serum albumin, 1 μg/μL of poly(deoxyinosinic-deoxycytidylic) acid sodium salt, 20% glycerol, and 2 mM dithiothreitol] with or without 10^−6^ M T_3_ and/or 10^−8^ M PCBs/dioxins for 30 min on ice. We added various amounts of control reticulocyte lysate to some samples to render a consistent total volume of lysate. After incubation, the samples were resolved by electrophoresis and visualized by autoradiography.

## Results

### Effects of PCBs/dioxins on TR-mediated transcription

**Figure 1:**
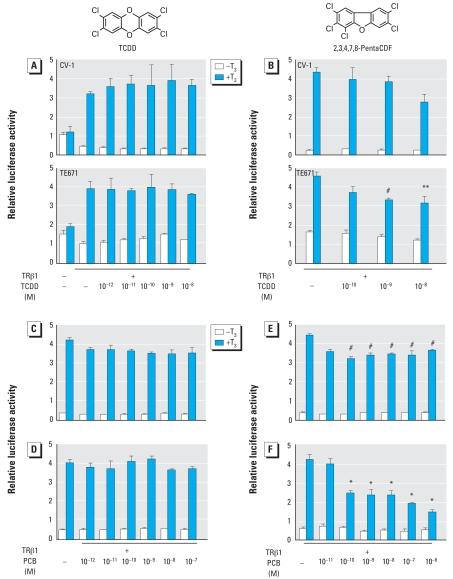
Effects of PCBs/dioxins on TR-mediated transcription (data represent mean ± SE of triplicates). (*A* and *B*) Expression plasmids encoding TRβ1 (10 ng) were cotransfected with F2-TK-LUC reporter plasmid (100 ng) into CV-1 and TE671 cells, and cells were incubated with or without 10^−7^ M T_3_ and indicated concentrations of TCDD (*A*) or pentaCDF (*B*). (*C*–*F*) Expression plasmids encoding TRβ1 (10 ng) were cotransfected with F2-TK-LUC reporter plasmid (100 ng) into CV-1 cells, and cells were incubated with or without T_3_ (10^−7^ M) and with indicated concentrations of PCB-114 (coplanar type; *C*), PCB-153 (*D*), 4-OH-PCB-165 (*E*), or 4-OH-PCB-106 (*F*). *p < 0.01, **p < 0.02, and ^#^*p* < 0.05 by ANOVA, compared with TRβ1 (+), T_3_ (+), and TCDD (−) in *A*, PCDF (−) in *B*, and PCB (−) in *C–F*.

**Figure 2:**
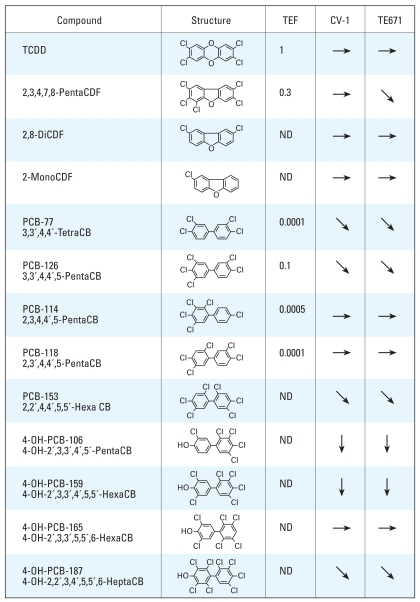
Effects of PCBs/dioxins on TR-mediated transcription in the presence of T_3_ (10^−7^ M) in CV-1 and TE671 cells. Down arrows indicate suppression > 50% at 10^−8^ M PCBs/dioxins; diagonal arrows indicate mild suppression (significant, but < 50% at 10^−8^ M); and right arrows represent no effect. TEF = 1 for the most toxic congener, TCDD. Congeners without TEF are indicated as ND (Van den Berg et al. 2006).

We previously reported that several PCB congeners, including OH metabolites such as 4-OH-PCB-106 and a PCB mixture (Aroclor 1254), suppress TR-mediated transcription (Iwasaki et al. 2002; Miyazaki et al. 2004). Here, we further investigated the effects of dioxins on TR-mediated transcription in monkey fibroblast-derived CV-1 and human medulloblastoma-derived TE671 cells using transient cotransfection experiments (Figure 1). The most toxic congener, TCDD (TEF = 1) (Van den Berg et al. 2006), in a range of concentrations from 10^−16^ to 10^−6^ M, did not suppress TR-mediated transcription activated by 10^−7^ M T_3_ (Figure 1A). TR-mediated transcription was weakly suppressed by 10^−9^ M pentaCDF (TEF = 0.3), which is relatively higher than the effective dose of 4-OH-PCB-106, in TE671 but not in CV-1 cells. Among the PCDF congeners, 2,8-diCDF and 2-monoCDF did not affect TR-mediated transcription (Figure 2).

We also examined the effects of several representative PCB congeners, including coplanar PCBs (Figure 1C), noncoplanar PCBs (Figure 1D), and OH-PCBs (Figure 1E,F). We selected these congeners on the basis of their *ortho*-substitution profiles and 4-OH-PCB metabolites, which had significant effects on TR (Iwasaki et al. 2002; Miyazaki et al. 2004). Both PCB-77 (non-*ortho*; Figure 2), a coplanar PCB congener, and PCB-153 (di-*ortho*; Figure 1D), a noncoplanar PCB, slightly suppressed TR-mediated transcription at 10^−10^ M and 10^−11^ M, respectively, whereas PCB-114 (mono-*ortho*; Figure 1C) and 4-OH-PCB-165 (Figure 1E) had no effects. On the other hand, 4-OH-PCB-106 (Figure 1F) effectively suppressed TR-mediated transcription. Figure 2 summarizes the results of the effects of PCB/dioxin congeners on TR, including those from our previous studies under the same experimental conditions using CV-1 and TE671 cell lines (Iwasaki et al. 2002; Miyazaki et al. 2004), with a distinct difference among these compounds. Hydroxylation, degree of chlorination, and structural coplanarity do not correlate with the magnitude of suppression of TR-mediated transcription.

### Correlation of suppression of TR action and TR-TRE dissociation

**Figure 3:**
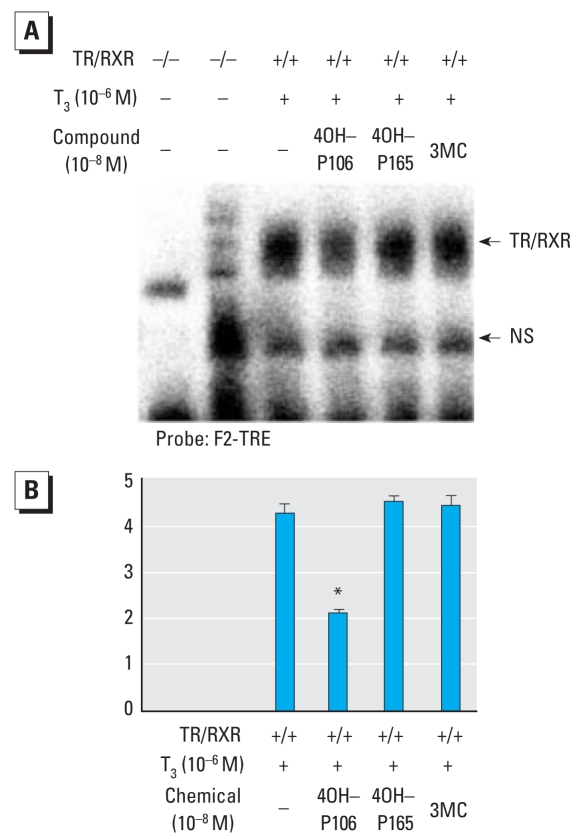
Magnitude of TR dissociation from TRE correlated with that of suppression by PCBs/dioxins. NS, nonspecific. (*A*) *In vitro* translated TRβ1 (1.5 μL) and/or RXRβ (3 μL) incubated with [^32^P]-labeled F2-TRE with or without 10^−6^ M T_3_ and 10^−8^ M 4-OH-PCB-106 (4OH-P106), 4-OH-PCB-165 (4OH-P165), or 3-methyl coranthrene (3MC). The results were similar in three independent repeats of the same experiment, and in experiments using DR4-TRE. (*B*) Histogram of relative intensity of dissociated TRβ1 from TRE by adding PCBs and 3MC. The intensity values of bands are ratios of intensity values with T_3_ without PCB/3MC. Results are mean ± SE of three independent experiments. *p < 0.01 by ANOVA and post hoc comparison using Bonferroni’s multiple range test compared with TR/RXR (+), T_3_ (+), and PCB (−).

We previously reported that suppression of TR-mediated transcription by 4-OH-PCB-106 is induced by partial dissociation of TR from TRE (Miyazaki et al. 2004). We examined the effects of PCBs/dioxins on TR-TRE binding using EMSA. Some of the PCB/dioxin congeners neither altered TR-mediated transcription nor affected TR-TRE binding. On the other hand, the PCB congeners that suppressed TR-mediated transcription effectively dissociated TR-TRE binding, as shown as a representative data in Figure 3. These results suggest that the magnitude of suppression of TR-mediated transcription by PCBs/dioxins correlates with those of the dissociation of TR-TRE binding, and that the site of action of PCBs in TR may be located within the DNA-binding domain (DBD).

### PCBs alter TR-mediated transcription through the DBD

**Figure 4:**
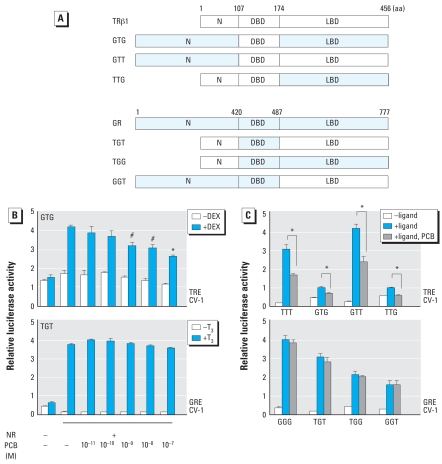
TR-mediated transcription altered by 4-OH-PCB-106 through TR-DBD. (*A*) Schematic structures and chimeric proteins used in the present study. Abbreviations: G, glucocorticoid receptor; N, N-terminal domain; NR, nuclear hormone receptor; T, thyroid hormone receptor. (*B*) Representative examples of PCB actions on chimeric receptor-induced transcription. Chimeric receptors (10 ng) were cotransfected with F2-TK-LUC or GRE-LUC reporter plasmid (100 ng) into CV-1 cells and incubated with or without T_3_ (10^−7^ M) or dexamethasone (DEX; 10^−7^ M) and 10^−11^–10^−7^ M 4-OH-PCB-106. (*C*) Effect of PCB on transcription through chimeric receptors containing TR-DBD or GR-DBD. Chimeric receptors (10 ng) were cotransfected with F2-TRE-LUC or GRE-LUC reporter plasmid (100 ng) into CV-1 cells and incubated with or without T_3_ (10^−7^ M) or DEX (10^−7^ M) and 10^−11^–10^−7^ M 4-OH-PCB-106. For B and C, data represent mean ± SE of triplicates. *p < 0.01, and ^#^*p* < 0.05 by ANOVA; for *B,* compared with GTG (+), DEX (+), and PCB (−).

Because several PCB congeners and their OH metabolites affect TR-mediated transcription by partially dissociating TR from TRE, we determined which functional domain of TR is affected by using 4-OH-PCB-106, which had the most suppressive effect among the PCB congeners and their OH metabolites. We previously showed that 4-OH-PCB-106 did not affect GR-mediated transcription (Iwasaki et al. 2002). We therefore constructed a series of chimeric receptors containing TR and GR functional domains (Figure 4A). Transcription of chimeric receptors containing TR-DBD was suppressed by 4-OH-PCB-106 (Figure 4B,C), whereas 4-OH-PCB-106 was not significantly suppressive when the chimeric receptors contained GR-DBD (Figure 4B,C). These results indicate that PCB congeners act on the TR through DBD rather than on the TR N-terminus or TR-ligand binding domain (LBD).

## Discussion

In the present study, we examined how PCBs/dioxins affect TR-mediated transcription and found distinct effects of several PCB congeners on TR. For example, several PCB congeners exerted significant suppression, whereas TCDD, the most toxic congener, did not. The magnitude of suppression was correlated with that of TR dissociation from TRE. Furthermore, we showed that these chemicals might act on TR-DBD.

We previously reported that the transcription mediated by TR is suppressed by 4-OH-PCB-106 (Iwasaki et al. 2002), and others have shown that dioxins and coplanar PCBs may disrupt the TH system (Bastomsky 1977; Gorski and Rozman 1987; Henry and Gasiewicz 1987; Kohn et al. 1996; Nishimura et al. 2002; Potter et al. 1986; Van Birgelen et al. 1995). Thus, we initially postulated that dioxins, coplanar PCBs, and OH-PCB compound suppress TR-mediated transcription. Although TCDD did not exert any effects, one PCDF congener and several PCB congeners, including mono-*ortho–*substituted congeners, suppressed TR action. On the other hand, 4-OH-PCB-165 had no suppressive effects. These results indicate that 4-hydroxylation and coplanarity are not essential for inducing the suppression.

We investigated which functional domain of TR is responsible for PCB action in a system using a series of chimeric receptors (Figure 4) and found that PCBs act on TR-DBD, but not on LBD. Although PCBs do not have high affinity for TR-LBD (Cheek et al. 1999), there are several possibilities for the interaction of PCBs and TR-DBD. It is conceivable that PCBs bind to and change the conformation of TR-DBD because we found that PCBs dissociate TR-coactivator complexes from TRE but not from TR-corepressor complexes (Miyazaki et al. 2004) and alter the binding between coactivators or corepressors and TR (Miyazaki et al. 2004). Coactivators bind to the activation function-2 domain of TR, which is located at the C-terminus of the LBD. In contrast, corepressors bind to a broad region of TRs, including the hinge region that is located immediately adjacent to the DBD. These observations are consistent with the notion that PCBs bind to the DBD and subsequently change the conformation of the domain and its surrounding region to induce the dissociation from TRE. Other possible interactions of PCBs and TR-DBD would be masking of the PCB-binding region of TR by corepressors and/or alteration of the TR-DBD conformation by PCBs binding or recruitment of a “PCB-responsive TR-binding protein.”

Although the present study revealed that PCBs suppress TR action, Gauger et al. (2007) found that some PCB congeners, such as PCB-105 and/or PCB-118 (mono-*ortho* PCB), may exert agonistic action toward TR-mediated transcription in rat somatomam-motroph-derived GH3 cells. Their study suggested that OH-PCB-105 or OH-PCB-118 may be responsible for this agonistic action toward TR because this action occurred when cytochrome P450 (CYP) expression was induced by PCB-126. Thus, a mixture of coplanar and noncoplanar PCBs might result in an agonistic effect on TR-mediated transcription. On the other hand, we confirmed by semiquantitative reverse transcriptase-polymerase chain reaction that CYP1A1 is not expressed in CV-1 cells and that various PCB congeners do not induce CYP1A1 expression [Supplemental Material, Figure 1S (available online at http://www.ehponline.org/members/2008/11176/suppl.pdf)]. Thus, we could refute the possible involvement of CYP1A1 and AhR in our experimental system, and the PCB congeners each might directly act on TR.

Dioxins/PCBs cause learning and memory impairment in children (Koopman-Esseboom et al. 1994) and in laboratory animals (Hojo et al. 2008; Schantz et al. 1996; Seo et al. 1999, 2000). However, the molecular mechanisms of PCB action in the brain have not been clarified. Because the amounts of AhR expressed in the brain are limited, AhR’s involvement in PCB/dioxin actions in the brain might be less than that in other organs where it is abundant, such as the liver and reproductive organs. Thus, other signaling pathways may be involved in the neurotoxic manifestations. A possible mechanism is the disruption of intracellular signaling pathways that depend on Ca^2+^ homeostasis (Kodavanti 2005; Simon et al. 2007). We have also shown that PCB congeners alter the intracellular Ca^2+^ levels in cultured neurons (Okada et al. 2005), which may be relevant to the altered expression of Ca^2+^ sensitive genes, such as c-*Jun* (Shimokawa et al. 2006). Another possible mechanism of neurotoxicity may be relevant to a decreasing trend in total T_4_ levels in people living in the general environment (Koopman-Esseboom et al. 1994; Nagayama et al. 1998). *In utero* and lactational exposures to TCDD have been reported to induce thyroid gland hyperplasia (Nishimura et al. 2003) or to induce the liver uridine diphosphate glucuronosyltransferase 1 family that catalyzes TH (Nishimura et al. 2005), which could induce hypothyroidism. However, the magnitude of decrease in brain TH levels might not be sufficient to induce hypothyroidism in the brain (Meerts et al. 2002). Instead, PCBs might suppress TR action in the brain by dissociating TR from TRE as shown in the present study, or act as agonists of TR in cells that express AhR and CYP1A1, as noted above (Gauger et al. 2007). Thus, we consider multiple pathways of PCB action to be involved in the induction of learning and memory disorders. Further analysis is required to clarify the distinct role of each of these possible signaling pathways.

In summary, we examined the effects of several representative PCB/dioxin congeners on TR-mediated transcription. We also generated chimeric receptors from TR and GR to identify the functional domain responsible for PCB action. Under our experimental conditions, PCBs apparently acted on the DBD of TR. We propose that several pathways should be considered to determine how PCB and its related compounds exert their toxic effects.
